# Unveiling the Nutraceutical and Nutricosmetic Potential of *Syzygium nervosum* Flower Buds: A Focus on Phytochemicals and In Vitro Bioactivities

**DOI:** 10.3390/molecules30081762

**Published:** 2025-04-15

**Authors:** Yan Liu, Limei Huang, Tingting Sun, Zhen Cao, Tao Feng, Huatian Wang, Min Sun, Heng Yue, Chuang Yu, Chuanwang Tong, Lingyun Yao, Wan Zhang

**Affiliations:** 1 School of Perfume and Aroma Technology, Shanghai Institute of Technology, Shanghai 201418, China; 2 State Key Laboratory of Food Science and Resources, Nanchang University, Nanchang 330047, China; 3 College of Food and Bioengineering, Wuhu Institute of Technology, Wuhu 241003, China; 4 Anhui Jiaotianxiang Biological Technology Co., Ltd., Xuancheng 242000, China

**Keywords:** *Syzygium nervosum*, phytochemical composition, antioxidant activity, enzyme inhibitory activity, molecular docking

## Abstract

The flower buds of *Syzygium nervosum* are traditional edible and medicinal plant materials for the treatment of inflammation and stomach disorders. With rising demand for natural products in food and cosmetics, the nutraceutical and nutricosmetic potential of the flower buds has been evaluated in this work. The antioxidant activity of ethanol and water extracts of *S. nervosum* flower buds were detected through free radical scavenging (DPPH, ·OH, and ABTS) assays, and their inhibitory effects on tyrosinase and elastase enzymes were also evaluated. The ethanol extract (SNEE) exhibited stronger antioxidant activity and superior inhibitory effects on both enzymes compared with the water extract (SNWE), highlighting its potential for anti-aging and skin-whitening applications. Meanwhile, the LC-QTOF-MS was employed for the identification of key chemical molecules responsible for the nutricosmetic properties. Moreover, the enzyme inhibitory mechanisms of the flower bud extracts were further elucidated using inhibition kinetics and molecular docking methods. This research underscores the promising nutraceutical and nutricosmetic potential of *S. nervosum* flower buds so as to offer important information for further developing the edible flower resource as skin feeding bioactive ingredients.

## 1. Introduction

Beauty from within is one of the best options in meeting the worldwide demand for pursuing healthy skin, which could be achieved via skin feeding from inside and outside through either topical or oral supplementation of functional constituents [1]. With the trend of promoting beauty from within, there has been a growing interest in developing food-derived components as nutraceuticals and nutricosmetics for feeding the skin [2]. Among the various food resources being explored, edible flowers have garnered great attention due to their unique sensory and nutritional characteristics, as well as their skin health benefits [3,4]. For example, the *Astragalus sinicus* flowers are rich in phenolics and flavonoids that could exert efficient antioxidant, anti-elastase, and anti-tyrosinase activities, which exhibit great potential for exploring nutraceutical and nutricosmetic constituents for skin health benefits [5].

*Syzygium nervosum*, formerly known as *Cleistocalyx operculatus*, is a plant species belonging to the Myrtaceae that is native to tropical/subtropical regions and has been traditionally used in Southeast Asian countries due to its muti-functional properties [6,7]. In some Asian countries, the flower buds of *S. nervosum* are a famous herbal medicine for the treatment of cold, fever, and inflammation [8,9]. In Vietnam and China, the flower buds have been traditionally consumed as tea and are often brewed in hot water to prepare a functional beverage for alleviating influenza and stomach disorders [10]. Besides the promising functionality of water-soluble constituents, ethanol extracts of the flower buds have also been found to possess many health benefits, such as antimicrobial, antioxidant, and anti-inflammatory bioactivities [11,12,13]. Furthermore, topical utilization of the flower bud extracts could improve skin conditions, including bruises, acnes, and skin ulcers [14]. These proven bioactivities suggest that *S. nervosum* flower buds could be developed as nutraceutical and nutricosmetic ingredients for skin feeding applications.

It is well-known that excessively generated reactive oxygen species (ROS) in the human body can cause oxidative-stress-related skin diseases, such as exacerbated skin aging and inflammation [15]. That is why antioxidant component dietary supplements usually have a positive influence on skin health [16]. Previously, several studies reported the antioxidant as well as enzyme inhibitory activities of *S. nervosum* flower bud extracts [6,17,18], while their specific bioactive molecules and corresponding action mechanisms have seldom been clarified. Based on exploration of new nutraceutical and nutricosmetic ingredients from natural resources, tyrosinase and elastase are the most commonly adopted enzymes for bioactivity screening due to their close association with skin aging and skin pigmentation [19,20]. Therefore, the inhibitory action and mechanism of *S. nervosum* flower bud extracts in relation to these enzymes should be explored in order to further elucidate the nutraceutical and nutricosmetic potential of the edible plant materials.

The food-derived constituents could provide a largely unexplored source for developing new functional ingredients for skin feeding [1,2]. In this work, the nutraceutical and nutricosmetic potential of *S. nervosum* flower bud extracts (water extract and ethanol extract) were evaluated through in vitro bioactivity assessments with a focus on the antioxidant and enzyme (tyrosinase and elastase) inhibitory capacities. Furthermore, the phytochemical profile of the flower buds was characterized through LC-QTOF-MS to identify the promising bioactive molecules and to further elucidate their action mechanism through molecular docking studies. This study could provide comprehensive insights into elucidating the nutraceutical and nutricosmetic potential of *S. nervosum* flower buds to pave the way for the innovative application of this edible flower resource.

## 2. Results and Discussion

### 2.1. Phytochemical Analysis of S. nervosum Flower Buds

The water extracts of *S. nervosum* flower buds (SNWE) are most frequently used in tea beverages, and the ethanol extracts of the flower buds (SNEE) are usually prepared for both oral and topical applications. Therefore, the extraction yield and phytochemical composition of the water and ethanol extracts were both detected. After freeze-drying, the SNWE had a higher extraction yield of dry weight materials (12.09%) compared to the SNEE (7.13%), which might be a result of the high content of water-soluble constituents contained in the flower buds (Table S1). The extraction yields’ result was similar to that of the *S. nervosum* flower buds obtained from the Red River Delta (Vietnam), and it was relatively higher than that of flower buds obtained from Ho Chi Minh City (Vietnam) [18,21]. In addition, variation in colors was observed for the two extracts (Figure S1), as the chemical composition and appearance of the plant extracts were influenced by the adopted extraction solvents [6,18]. Consequently, the total phenolic content (TPC) and total flavonoid content (TFC) exhibited obvious differences between the two extracts, with the TPC revealed to be 94.24 and 53.30 mg GAE/g and the TFC revealed to be 26.67 and 18.00 mg QE/g for the SNWE and the SNEE, respectively. The TPC and TFC of the *S. nervosum* flower bud extracts were slightly lower than reported values [18,21]. In addition to the known flavonoid compound 2′,4′-dihydroxy-6′-methoxy-3′,5′-dimethylchalcone (DMC), there is little information on elucidating the detailed molecule components of the flower buds [12,13]. Therefore, LC-QTOF-MS was subsequently introduced to further characterize the phytochemical profile of the *S. nervosum* flower buds.

**Figure 1:**
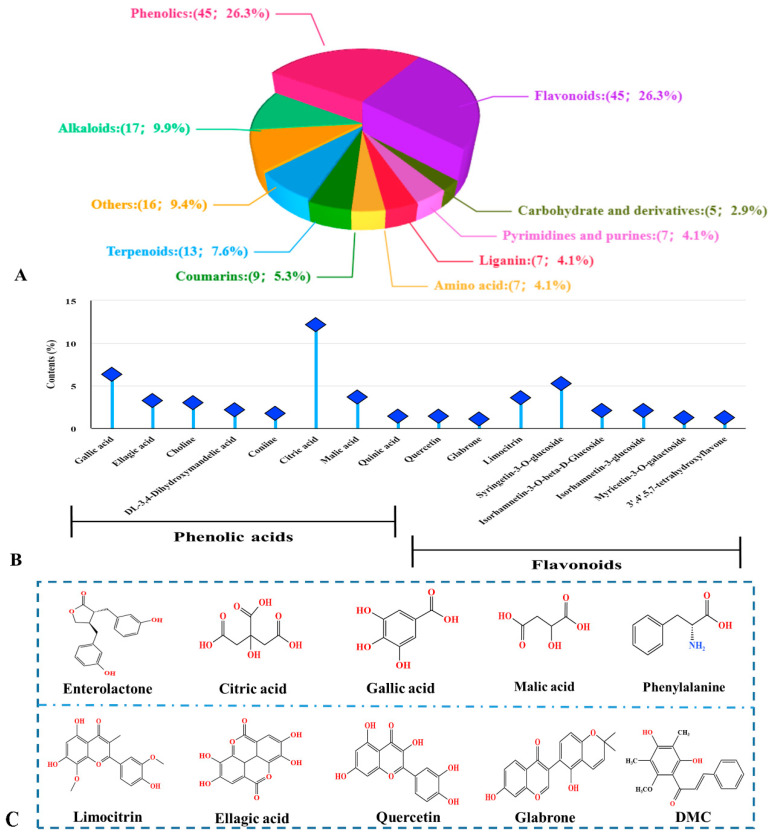
Phytochemical compounds in the *S. nervosum* extract. Distribution of 10 major compound classes (relative abundance, %) in the SNEE (**A**). Quantitative profile of major compounds (>1%) in phenolic acid and flavonoid groups (**B**). Chemical structures of ten representative compounds identified in the extract (**C**). The molecular structures were drawn using KingDraw v3.0 (KingDraw Chemical Software) based on data from ChemicalBook (https://www.chemicalbook.com/ProductChemicalPropertiesCB8328781_EN.htm, accessed on 7 April 2024). SNEE: *S. nervosum* ethanol extract.

A total of 171 compounds were identified in the ethanol extract of the *S. nervosum* flower buds, including flavonoids, phenolics, amino acids, alkaloids, coumarins, lignans, terpenoids, other substances, etc. (Figure 1, Figure S3 and Table S2). For the identified compounds, the most abundant components were flavonoids (such as quercetin, glabrone, and limocitrin) and phenolic acids (such as gallic acid, ellagic acid, and other organic acids) (Figure 1B). The result here was consistent with previous observations, which demonstrated that the flower buds of *S. nervosum* were mainly composed of flavonoids, phenolics, and triterpenoids that might contribute to their various pharmacological activities [6,12,13]. Flavonoids and phenolic acids are well-known antioxidant molecules in plant materials due to the phenolic hydroxyl groups presented in their structure [22], suggesting that the flower bud extracts of *S. nervosum* could serve as potential antioxidant ingredients used for nutraceutical and nutricosmetic products. Based on the content of identified phytochemicals through LC-QTOF-MS (Table S2) and the proven bioactive components in the flower buds of *S. nervosum*, 10 major compounds were assumed to be the most promising compounds that contribute to the nutraceutical and nutricosmetic properties of the flower bud extracts (Figure 1C).

### 2.2. In Vitro Antioxidant Activity of the Flower Bud Extracts

**Figure 2:**
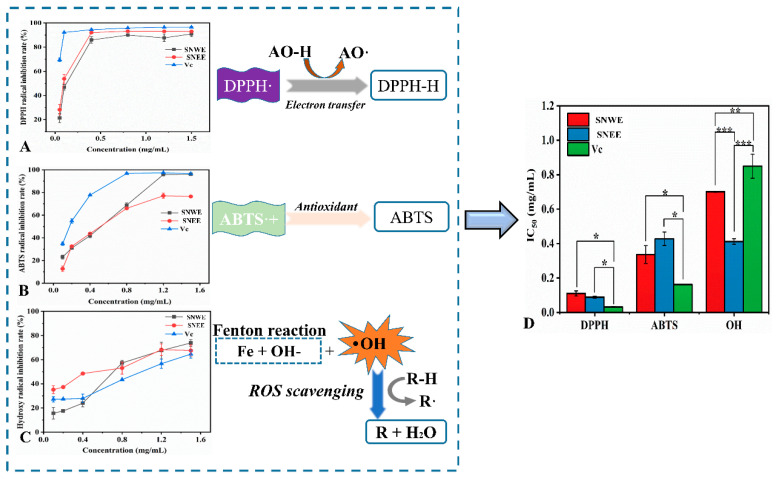
The inhibitory effects and mechanisms of the *S. nervosum* extracts on free radicals. DPPH radical inhibition rates (**A**). ABTS radical inhibition rates (**B**). Hydroxy radical inhibition rates (**C**). IC_50_ value (**D**). * indicates significance at *p* ≤ 0.05. ** indicates significance at *p* ≤ 0.01. *** indicates significance at *p* ≤ 0.001. SNEE: *S. nervosum* ethanol extract. SNWE: *S. nervosum* water extract. Vc: ascorbic acid.

The in vitro antioxidant activities of the SNEE and the SNWE were evaluated through DPPH, ABTS, and hydroxyl free radical scavenging assays. As shown in Figure 2, each sample showed concentration-dependent scavenging of the three free radicals. The SNEE exhibited a stronger DPPH free radical scavenging rate (IC_50_ = 0.07 mg/mL) compared to the SNWE (IC_50_ = 0.11 mg/mL), and the IC_50_ values of both samples were lower than that of the positive control, vitamin C (IC_50_ = 0.03 mg/mL) (Figure 2A). The results indicated that the two samples might exhibit strong electron transfer or hydrogen atom donation capabilities, making them effective in neutralizing DPPH radicals (Figure 2A). Similar results were also observed in previous work, which suggested that ethanol extract of the *S. nervosum* flower buds exhibited the strongest antioxidant activity among the seven selected extraction solvents [18]. The scavenging capacity of the SNWE against the ABTS free radicals was found to closely resemble that of the SNEE under a concentration range of 0.1–0.8 mg/L, and it exhibited more efficient antioxidant activity than the SNEE upon increased concentration (Figure 2B). In addition, the hydroxyl free radical scavenging assay indicated that IC_50_ was revealed to be 0.41, 0.70, and 0.84 for the SNWE, the SNEE, and the positive control (vitamin C), respectively, which suggested the significant (*p* < 0.05) potential of the flower bud extracts against the ·OH radicals due to the lower IC_50_ values (Figure 2C).

### 2.3. Enzyme Inhibitory Activity of the Flower Bud Extracts

#### 2.3.1. Tyrosinase and Elastase Inhibitory Assays

**Figure 3:**
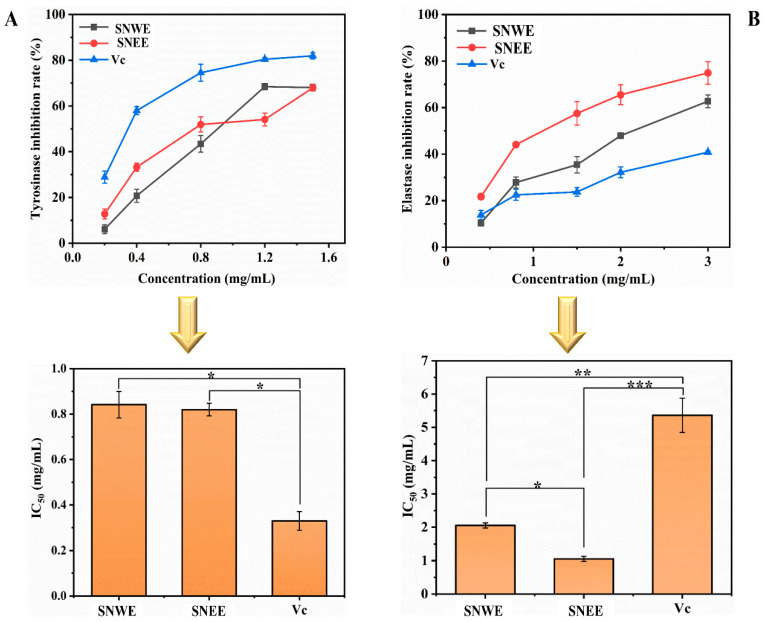
Tyrosinase inhibitory activity (**A**) and elastase inhibitory activity (**B**) of the *S. nervosum* extracts. * indicates significance at *p* ≤ 0.05. ** indicates significance at *p* ≤ 0.01. *** indicates significance at *p* ≤ 0.001. SNEE: *S. nervosum* ethanol extract. SNWE: *S. nervosum* water extract.

Melanin plays an important role in maintaining skin health through the multiple-step transformation of L-tyrosine, while abnormal accumulation of melanin in the human body could lead to various skin disorders, such as hyperpigmentation, freckles, and skin cancer [5]. Therefore, tyrosinase inhibitors are often the most commonly employed method in treating melanin-related skin health. The water and ethanol extracts of *S. nervosum* flower buds both revealed excellent tyrosinase inhibitory activities, with the IC_50_ values determined to be 0.84 and 0.82 mg/mL for the SNWE and the SNEE, respectively (Figure 3A). As reported elsewhere, the tyrosinase inhibitory effect of the bioactive component might be attributed to its significant antioxidant activity, such as reducing agents (vitamin C and cysteine), which can inhibit melanogenesis by converting dopaquinone back to L-dopa and therefore possess skin-whitening properties [23]. The SNWE and the SNEE have exhibited both excellent antioxidant and tyrosinase inhibitory capacities, suggesting that *S. nervosum* flower buds might serve as a novel reducing agent with great potential applications in nutraceutical and nutricosmetic ingredients.

Elastase belongs to the chymotrypsin family of proteases, and it is responsible for the degradation of elastin as well as other proteins that are fundamental for forming a supportive framework and maintaining the elasticity of the skin [19]. The excessive hydrolysis of the dermal elastin is strongly correlated to skin aging and wrinkles, so elastase inhibitors are often adopted as anti-wrinkle agents for promoting the preservation of skin elasticity [5]. As shown in Figure 3B, the SNEE exhibited the highest elastase inhibitory activity (IC_50_ = 1.05 mg/mL), which was significantly (*p* ≤ 0.05) greater than that of the SNWE (IC_50_ = 2.05 mg/mL) and the positive control (IC_50_ = 5.36 mg/mL). For human skin, elastase can be activated by reactive oxygen species (ROS) and causes extrinsic skin aging circumstances, suggesting that the antioxidant components might also serve as promising anti-aging ingredients [19]. The efficient antioxidant and anti-elastase capacity of the extracts observed (Figure 2 and Figure 3B) suggested that *S. nervosum* flower buds revealed great potential for developing new anti-aging and anti-wrinkle agents. The enzyme inhibitory activities of the *S. nervosum* flower buds on α-glucosidase and urease have been well-clarified [6,18], while its effects on tyrosinase and elastase have seldom been reported in previous work. In order to better understand its tyrosinase/elastase inhibitory mechanism, the enzyme inhibition kinetics of the SNEE were further evaluated.

#### 2.3.2. Enzyme Inhibition Kinetics Analysis

**Figure 4:**
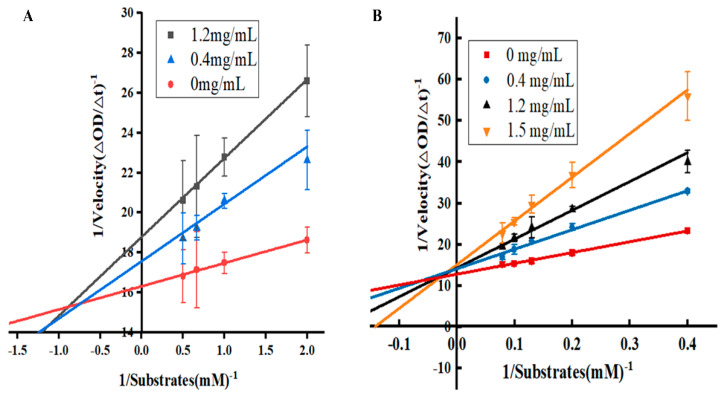
Lineweaver-Burk plots of elastase inhibitory activity (**A**) and tyrosinase inhibitory activity (**B**) of SNEE. SNEE: *S. nervosum* ethanol extract.

Enzyme inhibitors are typically classified into four categories: competitive, non-competitive, mixed (competitive–uncompetitive), and uncompetitive inhibition [24]. Here, the Lineweaver–Burk plots were employed to investigate the enzymatic inhibitory mechanism of the flower bud extracts. For the elastase inhibitory experiments under a constant concentration of both substrate and elastase, the enzyme reaction rate decreased with the improved concentration of the SNEE (Figure 4A), suggesting a reduction in the catalytic efficiency of elastase. As shown in Figure 4A, the straight lines intersected in the second quadrant, suggesting a mixed inhibition (competitive and uncompetitive) effect of the SNEE on elastase, which indicated the binding capacity of inhibitors to free enzymes and enzyme–substrate complexes [5,24]. In the case of tyrosinase, the slope of the plot increased with the improved concentration of the SNEE, followed by the decreased value in maximum velocity (Vmax) (Figure 4B). Plotting the reciprocal of substrate concentration (1/[S]) against the reciprocal of the enzymatic reaction rate (1/velocity) obtained three converging straight lines in the second quadrant (Figure 4B), which also indicated that the SNEE could inhibit the reaction via a mixed competitive–uncompetitive inhibition mechanism due to its capability of binding to both the free enzyme and the enzyme–substrate complex. These findings suggest that the SNEE could serve as a promising tyrosinase/elastase enzyme inhibitor due to its contained phytochemicals, which would offer a bright future in nutraceutical and nutricosmetic applications [5].

### 2.4. The Correlation Between Phytochemical Composition and Bioactivity

The Pearson correlation analysis was employed to evaluate the correlation between the bioactivity (antioxidant and anti-enzymatic activities) and the phytochemical composition (TPC and TFC) of the flower bud extracts [5,25]. As shown in Table 1, the correlation coefficients for the corresponding horizontal and vertical measurements were identified in each cell situated in the upper-right section of the table. The results revealed a positive correlation between the bioactivities of the flower bud extracts and their TPC or TFC values. Specifically, significant correlations were found between TPC and DPPH (r = 0.89, *p* ≤ 0.05) as well as ·OH (r = 1.00, *p* ≤ 0.001) free radical scavenging capacities (Table 1). Similarly, the correlation coefficient for TFC showed a conspicuous trend, which revealed significant (*p* ≤ 0.05) positive correlations with DPPH (r = 0.87) and ·OH (r = 1.00), respectively (Table 1). These results align with those of previous studies that have highlighted a strong relationship between the flavonoid/phenolic compounds and antioxidant activities [5,25]. However, no significant correlation was observed between TPC/TFC and ABTS inhibitory activity (*p* > 0.05), suggesting that other undetected components might contribute to the ABTS radical scavenging activity. Meanwhile, the correlation analysis revealed only a weak association between the TPC/TFC and tyrosinase inhibitory activity (*p* > 0.05), while a strong significant relationship was found between the TFC/TPC and elastase inhibitory activity (*p* ≤ 0.001) (Table 1). Interestingly, elastase inhibitory activity also showed a strongly significant correlation with DPPH and ·OH inhibitory activities of the extracts (*p* ≤ 0.001), consistent with previous findings suggesting a positive link between anti-elastase activity and radical scavenging capabilities [19]. Robust antioxidant activity plays a crucial role in maintaining skin health and tissue elasticity by neutralizing free radicals that accelerate skin aging and compromise its health [1]. The results underscore the positive contributions of both phenolic and flavonoid content on the antioxidant and elastase inhibition capacity of the flower bud extracts. It is obvious that not all of the flavonoid/phenolic compounds could exhibit strong inhibitory effects on tyrosinase and elastase; hence, the TPC/TFC values might not adequately elucidate the enzyme inhibitory capacity of the flower buds. To address this, the effect of each identified compound (Figure 1C) on anti-tyrosinase and anti-elastase activity, as well as its specific inhibitory mechanism, were further evaluated using the molecular docking method in subsequent work.

### 2.5. Molecular Docking Analysis

**Figure 5:**
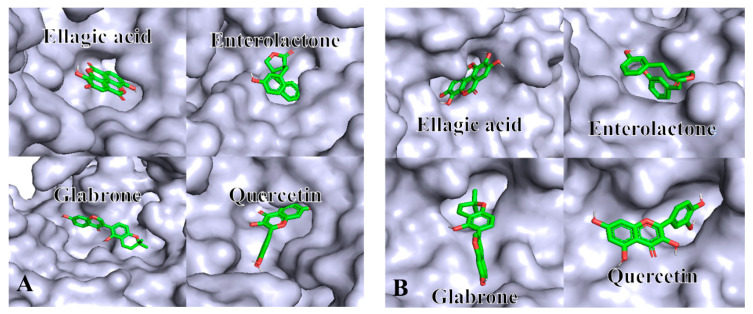
The docking model of the enzymes to major phenolic compounds. The 3D binding mode of the active sites of tyrosinase (**A**) and elastase (**B**) to ellagic acid, enterolactone, glabrone, and quercetin.

Molecular docking, a widely used computational simulation technique, is frequently employed to evaluate the binding modes and relative affinities in protein–ligand interactions [26]. In this study, the binding energies for the enzyme–substrate interactions of 10 selected compounds with the target enzymes were calculated (Table 2). The results indicated that all interactions were spontaneous, as the binding energies of all dockings were all lower than 0 [27]. Notably, quercetin and ellagic acid exhibited strong affinities for both enzymes, with binding energies below −7 kcal/mol. Similarly, enterolactone and glabrone also demonstrated significant interactions with tyrosinase (−7.6 kcal/mol) and elastase (−7.5 kcal/mol). Consequently, ellagic acid, enterolactone, glabrone, and quercetin were selected as representative ligands for further investigation through both 3D and 2D docking studies. Based on the 3D molecular docking results (Figure 5A,B), both enzymes exhibited flexible regions that facilitated the entry of small molecules into their interior through pores or flexible regions near surface cavities. Previous research has shown that multiple hydrogen bonds within the internal cavities of enzymes could strengthen the stability of ligand–receptor complexes [28]. For quercetin, in the 2D plot, three hydrogen bond interactions were observed with tyrosinase residues His-85, His-263, and Ser-282, and four hydrogen bonds were formed with Ser-190, Asn-192, Ser-214, and Gly-216 residues of elastase (Figure S2 and Table 3). The hydrogen bonds were probably linked to the presence of hydroxyl groups at the binding site of enzymes and quercetin molecules in an aqueous environment. Nonetheless, glabrone primarily established an unfavorable donor–donor interaction, along with a few π–sigma interactions with tyrosinase (Figure S2). These interactions might not favor ligand–enzyme binding, which could explain the greater binding energy observed for tyrosinase compared to other active compounds. Consequently, hydrogen bonds might be the primary force responsible for forming a stable binding conformation, thereby enhancing the inhibition of enzyme activity. The molecular docking results indicated that quercetin and ellagic acid exhibited strong association with both enzymes (tyrosinase and elastase) with higher numbers of hydrogen bonds formed, highlighting their potential as promising inhibitors of the two enzymes (Table 3). In addition, enterolactone can form a strong complex with tyrosinase specifically, while glabrone exhibited a firm binding affinity for elastase (Table 3). All of these results indicated that the *S. nervosum* flower buds revealed promising potential in developing newly plant-derived nutraceutical and nutricosmetic ingredients for functional food and cosmetic products. However, further investigations, such as in vivo and toxicology experiments, are still required to confirm the specific efficacy and safety of the flower bud extracts.

## 3. Future Perspectives

The findings from this study open the door to the potential development of cosmetic products derived from *S. nervosum*. Given the observed bioactivities, future work could focus on formulating either a topical or oral cosmetic product, depending on the desired effect and target application. A topical formulation might be more suitable for skin care applications aiming to improve skin hydration, anti-aging, etc., while an oral product could target systemic benefits, such as antioxidant support or skin health from within.

Several challenges must be addressed in the development process, such as the stability of the active ingredients in both formulations, the appropriate concentration for effective use, and ensuring safety and non-toxicity for consumers. Additionally, the scalability of the production process for commercial purposes will need to be evaluated.

Future steps of our study will include in vitro testing using established cell lines to assess the bioactivity and safety of the extracts, followed by preclinical animal studies to confirm efficacy and tolerability. Depending on the results, clinical trials in humans may be necessary to further evaluate the product’s performance and safety in a real-world context.

## 4. Materials and Methods

### 4.1. Plant Materials and Reagents

The dried flower buds of *S. nervosum* were purchased from an online e-commerce platform (www.taobao.com), which collected them from Liuzhou (Guangxi, China) in June 2023. The buds were ground into powder (60-mesh) and stored in a sealed bag at −20 °C until use. The 2,2′-azino-bis (3-ethylbenzothiazoline-6-sulfonic acid) ammonium salt (ABTS) and 2,2-diphenyl-1-picrylhydrazyl (DPPH) were purchased from Sigma-Aldrich (Shanghai, China). Tyrosinase and elastase enzymes were purchased from Shanghai Boer Chemical Reagents Co., Ltd. N-succinyl-Ala-Ala-Ala-p-nitroanilide was purchased from Gaoxin Glass Instrument Co., Ltd. (Shanghai, China). All other reagents are analytical-grade and purchased from Sinopharm Chemical Reagent Co., Ltd. (Shanghai, China).

### 4.2. Preparation of the Flower Bud Extracts and Phytochemical Measurements

Dried powders (10 g) of the flower buds were individually ultrasonic extracted (25 kHz, 150 w) three times with 200 mL of ethanol (90%) or water each for 30 min at 40 °C. Afterward, the mixture was filtered to separate the liquid extract. The remaining ethanol or water was then removed using a rotary evaporator under reduced pressure (RE 52-86A, Shanghai Yarong Biochemical Instrument Factory, Shanghai, China) and further freeze-dried to obtain the ethanol extract (SNEE) and the water extract (SNWE) of the *S. nervosum* flower buds (Figure S1). For phytochemical composition analysis, the total phenolic content (TPC) of each extract was assessed using the Folin–Ciocalteu method, as reported elsewhere [5]. Briefly, 2 mL of 0.2 N Folin-Ciocalteu reagent was added to a tube containing 500 μL of the redissolved extract. After fully mixing, the mixed solution was added to 1 mL of saturated sodium carbonate solution and kept in the dark at room temperature for 90 min. Then, the absorbance of the mixture was measured at 765 nm using a SkyHigh microplate reader (Thermo Fisher Scientific Inc., 81 Wyman Street, Waltham, MA, USA) with gallic acid used as the standard, and the TPC was expressed as milligrams of gallic acid equivalents (GAE)/g freeze-dried extract. In addition, the total flavonoid content (TFC) was quantified using a spectrophotometric method reported previously, with quercetin as the standard [5]. Firstly, 4 mL of each sample solution was mixed with 4 mL of a 2% AlCl_3_–methanolic solution and incubated in the dark at room temperature for 15 min. Then, the absorbance of the mixture was recorded at 430 nm using the microplate reader, and the total flavonoid content (TFC) was expressed as milligrams of quercetin equivalents (QE)/g freeze-dried extract.

### 4.3. LC-QTOF-MS Analysis

The LC-QTOF-MS analysis was performed to characterize the phytochemical profile of the *S. nervosum* flower buds using an Agilent 1290 Infinity UPLC coupled to an Agilent 6545 QTOF mass spectrometer equipped with an electrospray ionization interface (Agilent Technologies, Santa Clara, CA, USA). Separation via LC was achieved using an ACQUITY UPLC HSS T3 column (2.1 × 100 mm, 3.5 μm particle size, Waters, Milford, CT, USA) with the temperature maintained at 40 °C. The mobile phase consisted of water (A) and acetonitrile (B), both containing 0.1% formic acid and 2 mM ammonium formate, and the elution gradient procedure was programmed as follows: 0 min, 95% A, 1.5 min, 95% A, 2.5 min, 90% A, 14 min, 60% A, 22 min, 5% A, 25 min, 5% A, 26 min, 95% A, and 30 min, 95% A. The injection volume was 5 μL, and the flow rate was maintained at 0.4 mL/min.

MS detection was conducted in both positive and negative ionization modes. Nitrogen served as the nebulization gas at a flow rate of 8.0 L/min with a capillary voltage of 4 kV and a drying gas temperature of 320 °C. The sheath gas was maintained at 320 °C with a flow rate of 12.0 L/min. MS accurate mass spectra were acquired within the range of 50–1300 *m*/*z* at a rate of 5 spectra per second. MS data processing was performed using MSDIAL 4.60 software.

### 4.4. Antioxidant Capacity Analysis of the Flower Bud Extracts

#### 4.4.1. DPPH Radical Scavenging Capacity

The DPPH radical scavenging capacity of the *S. nervosum* flower bud extracts was determined using a method reported previously, with slight modifications [5]. As usual, 50 μL of a DPPH solution (0.1 mM) prepared in ethanol was added to 150 μL of each tested sample. The mixture was vortexed and incubated in the dark for 30 min before measuring the absorbance at 517 nm at room temperature. Ethanol and distilled water were used as the control and the blank, respectively. The DPPH radical scavenging activity of the extracts was quantified using Equation (1):

Radical scavenging rate (%) = [1 − (Ai − Aj)/A0] × 100%(1)

where A0 represents the absorbance of the blank, Ai represents the absorbance of the sample, and Aj represents the absorbance of the control.

#### 4.4.2. Hydroxyl Radical Scavenging Capacity

The hydroxyl radical scavenging capacity of each sample was measured according to Cai’s method, with minor modifications [5]. The sample solution was mixed with 1 mL of 6 mM H_2_O_2_ solution, 1 mL of 6 mM FeSO_4_, and 1 mL of 6 mM salicylic acid–ethanol. The mixture was shaken well and incubated at 37 °C for 30 min before being detected at an absorbance of 510 nm using the microplate reader. The control was prepared with solvent instead of salicylic acid–ethanol, while solvent replaced the sample solution for the blank. The hydroxyl radical scavenging rate was calculated the same way it was calculated using Equation (1).

#### 4.4.3. ABTS Radical Scavenging Capacity

The ABTS radical scavenging capacity of each sample was measured using Yao’s method, with slight modifications [25]. Briefly, the ABTS working solution was prepared using ABTS solution (7 mM) and potassium persulphate (2.45 mM) solution, and the anhydrous ethanol was used to dilute the solution when the absorbance reached 0.7 ± 0.02 at 734 nm. Subsequently, the sample solution (1 mL) was mixed with the diluted working solution (3 mL) and allowed to stand for 10 min at room temperature. The mixtures were detected at 734 nm. The blank was prepared with solvent instead of the samples, and the control was prepared using ethanol instead of the diluted working solution. The ABTS radical scavenging rate was calculated according to Equation (1).

### 4.5. Tyrosinase and Elastase Inhibitory Activity of the S. nervosum Flower Bud Extracts

The inhibitory effect of each sample on the enzyme activity of mushroom tyrosinase was measured using L-DOPA as the substrate, as previously described [5]. Briefly, a mixture consisting of 100 μL of tyrosine (150 U/mL), 180 μL of PBS buffer (0.2 M, pH 6.8), and 10 μL of sample was incubated at 37 °C for 10 min. Following this, 50 μL of L-DOPA solution (2 mM) was added to the mixture and incubated at 37 °C for an additional 5 min. The absorbance of the final solution was promptly recorded at 475 nm using the microplate reader, and the tyrosinase inhibition rate was then determined using the following Equation (2):

Enzyme inhibition rate (%) = [1 − (Aj − Ak)/(A0 − Ai)] × 100%(2)

where A0 represents the absorbance of the sample blank that uses buffer solution instead of the sample, Ai represents the absorbance of the substrate and sample blank that uses buffer solution instead of both substrate and the sample, Aj represents the absorbance of the tested sample, and Ak represents the absorbance of the tyrosinase blank that uses buffer solution instead of tyrosinase.

The elastase inhibitory potential of the extracts was evaluated based on the method described previously, with slight modifications [5]. Briefly, each sample of different concentrations was mixed with 0.10 mL of enzyme solution and 2 mL of 0.15 M Tris-HCl buffer (pH 8.0) and kept at room temperature for 15 min. Subsequently, 0.20 mL of the substrate solution (N-succinyl-Ala-Ala-Ala-p-nitroanilide in buffer) was added to the mixture and reacted at room temperature for 10 min, and the absorbance was measured at 410 nm. Meanwhile, the sample blank and the elastase blank were prepared similarly to the tyrosinase inhibition detections, and the elastase inhibitory activity was calculated using Equation (2).

### 4.6. Enzyme Inhibition Kinetics of the Flower Bud Extracts

The enzyme inhibition kinetics were employed to evaluate the inhibition type and mechanism of the *S. nervosum* flower bud extracts. Reactions were conducted using different concentrations of enzyme (tyrosinase and elastase) and of the flower bud sample solution, with the enzymatic reaction rate V expressed as absorbance change (ΔOD) [5]. For tyrosinase inhibition, experiments were conducted in a 96-well plate at 37 °C; to each well was added 50 μL of substrate L-DOPA (0–2.5 mg/mL), 50 μL of the sample solution (flower bud extracts), and 100 μL of a tyrosinase aqueous solution (150 U/mL). After reacting for 5 min, absorbance was promptly measured at 475 nm using a microplate reader. For elastase inhibition kinetics, elastase (50 μL) was firstly mixed with 100 μL of sample solution (0–1.2 mg/mL) and incubated at 25 °C for 2 min. Then, 50 μL of varying concentrations of the substrate (N-succinyl-Ala-Ala-Ala-pNA) was added, and the mixture was incubated at 25 °C for 5 min. Thereafter, the absorbance of the solution at 410 nm was measured using a microplate reader. The type of enzyme inhibition was determined by analyzing the inverse Lineweaver–Burk plot of the reaction velocity (1/V) against substrate concentration (1/[S]).

### 4.7. Molecular Docking

Molecular docking was introduced to predict the main bioactive compounds, as well as their action mechanisms, of the *S. nervosum* flower bud extracts using Autodock Vina 1.2.5, with the most abundant components presented in the extracts used as the docking ligands and two enzymes (tyrosinase and elastase) used as receptor targets. Firstly, the structures of the tyrosinase (PDB ID: 2Y9X) and the elastase (PDB ID: 1BRU) were imported into Chem3D software (Version 22.0.0) to obtain the minimized energy form and saved in PDB format. The three-dimensional (3D) structures of the tyrosinase and elastase were downloaded from the RCSB Protein Data Bank (http://www.rcsb.org/, accessed on 6 July 2024). The docking input files of both proteins and ligands were generated using the AutoDockTools 1.5.6 package. The search grid parameters of the tyrosinase and the elastase were retrieved from previously reported work [26,29].

### 4.8. Statistical Analysis

All measurements were conducted in triplicate. Unless otherwise specified, data are presented as the mean ± standard deviation (SD). Statistical comparisons were performed using Duncan’s test following a one-way analysis of variance (ANOVA) in SPSS software (27.0.1, SPSS Inc., Chicago, IL, USA). Statistical significance was determined at a level of *p* ≤ 0.05. The Pearson correlation analysis was conducted to evaluate the relationships between total phenolic content, the flavonoid content of the extracts, and their respective radical and enzyme inhibition activities, and the results were visualized using Origin 2022 software (Origin 2022, OriginLab Corporation, Northampton, MA, USA).

## 5. Conclusions

In this study, we systematically explored the phytochemical composition, antioxidant properties, and enzyme inhibitory activities and mechanisms of *S. nervosum* flower buds extracted using water and ethanol. Our results revealed that the water extract (SNWE) demonstrated higher total phenolic and flavonoid content, whereas the ethanol extract (SNEE) exhibited superior antioxidant capacity in DPPH and hydroxyl radical scavenging assays. Additionally, the SNEE showed significant tyrosinase and elastase inhibitory activities, suggesting its potential for anti-aging and skin-whitening applications. The molecular docking analysis further confirmed the strong binding interactions of key phenolic compounds, such as quercetin and ellagic acid, with tyrosinase and elastase, reinforcing the bioactivity of these extracts. Overall, this study highlights the nutraceutical and nutricosmetic potential of *S. nervosum* extracts, particularly the ethanol extract, as a source of bioactive compounds with promising applications in functional food and cosmetics formulations.

## Figures and Tables

**Table 1:** The Pearson correlation coefficients between total phenolic content (TPC), total flavonoid content (TFC), antioxidant activity (DPPH, ABTS, OH), elastase inhibition (EIA), and tyrosinase inhibition (TIA) in *S. nervosum* flower buds.

	TPC	TFC	DPPH	ABTS	OH	TIA^a^	EIA^b^
TPC	1.00	0.92 **	0.89 *	0.44	1.00 ***	0.40	1.00 ***
TFC		1.00	0.87 *	0.39	1.00 ***	0.39	0.99 ***
DPPH			1.00	0.70	0.90 *	0.58	0.91 *
ABTS				1.00	0.45	0.15	0.47
OH					1.00	0.38	1.00 ***
TIA						1.00	0.41
EIA							1.00

TIA^a^: tyrosinase inhibitory activity, EIA^b^: elastase inhibitory activity. * indicates significance at *p* ≤ 0.05. ** indicates significance at *p* ≤ 0.01. *** indicates significance at *p* ≤ 0.001.

**Table 2:** Binding energy (kcal/mol) of tyrosinase and elastase with ten major compounds present in SNEE. SNEE: *S. nervosum* ethanol extract.

No.	Compounds	Binding Energy (kcal/mol)	
		Tyrosinase	Elastase
1	Citric acid	−5.3	−5.0
2	Ellagic acid	−7.2	−7.5
3	Enterolactone	−7.6	−6.6
4	Gallic acid	−6.0	−5.8
5	Glabrone	−6.8	−7.5
6	Limocitrin	−6.7	−7.0
7	Malic acid	−4.9	−4.5
8	Phenylalanine	−5.8	−4.9
9	Quercetin	−7.9	−7.5
10	DMC	−6.4	−6.9

**Table 3:** Binding results of enzymes with ellagic acid, enterolactone, glabrone, and quercetin, respectively.

**Hydrogen** **bonding**	**Docking Conformations**	**Residues**	**Amino Acid**
	Tyrosinase–ellagic acid complex	85 (A chain)	His
		244 (A chain)	His
	Tyrosinase–enterolactone complex	268 (A chain)	Arg
	Tyrosinase–quercetin complex	85 (A chain)	His
		263 (A chain)	His
		282 (A chain)	Ser
	Elastase–ellagic complex	57 (P chain)	His
		191 (P chain)	Cys
		192 (P chain)	Asn
		195 (P chain)	Ser
	Elastase–glabrone complex	96 (P chain)	Ser
		195 (P chain)	Ser
		216 (P chain)	Gly
	Elastase–quercetin complex	190 (P chain)	Ser
		192 (P chain)	Asn
		195 (P chain)	Ser
		214 (P chain)	Ser

## Data Availability

Data are available upon request.

## Supplementary Materials

The following supporting information can be downloaded at https://www.mdpi.com/article/10.3390/molecules30081762/s1, Figure S1: The schematic diagram of various solvent extraction processes applied to *Syzygium nervosum* flower buds; Figure S2: The 2D schematic interaction diagram between ellagic acid, enterolactone, glabrone, and quercetin and the active amino acid residues of the tyrosinase (A) and elastase (B); Figure S3: The total ion current (TIC) chromatogram of *Syzygium nervosum* extracts. Table S1: Total phenolic and flavonoid contents of *Syzygium nervosum* extracts; Table S2: Phytochemical compounds identified in the ethanol extract of *S. nervosum* flower buds via LC-Q-TOF-MS.
