# Supplementary material for: Unveiling the Nutraceutical and Nutricosmetic Potential of Syzygium nervosum Flower Buds: A Focus on Phytochemicals and In Vitro Bioactivities
Source: Molecules. 2025 Apr 15;30(8):1762. doi: 10.3390/molecules30081762 (PMC12029419; doi:10.3390/molecules30081762)
Supplement: Supplementary file 1 [file molecules-30-01762-s001.zip › molecules-3570782-supplementary.pdf]

Unveiling the nutraceutical and nutricosmetic potential of Syzygium nervosum flower buds: A focus on phytochemicals and in-vitro bioactivities

Yan Liu[1,#], Limei Huang[1,#], Tingting Sun[1], Zhen Cao[2], Tao Feng[1], Huatian Wang[1], Min Sun[1,*], Heng Yue[1], Chuang Yu[1], Chuanwang Tong[3], Lingyun Yao[1], Wan Zhang[4,*]

1 School of Perfume and Aroma Technology, Shanghai Institute of Technology, Shanghai 201418, China.

2 State Key Laboratory of Food Science and Resources, Nanchang University, Nanchang 330047, China

3 College of Food and Bioengineering, Wuhu Institute of Technology, Wuhu 241003, China.

4 Anhui Jiaotianxiang Biological Technology Co., Ltd, Anhui, China

*** Correspondence: Min Sun, sunmin@sit.edu.cn;**

Wan Zhang, zw20220904@sina.com

# These two authors have made equal contributions to this work.

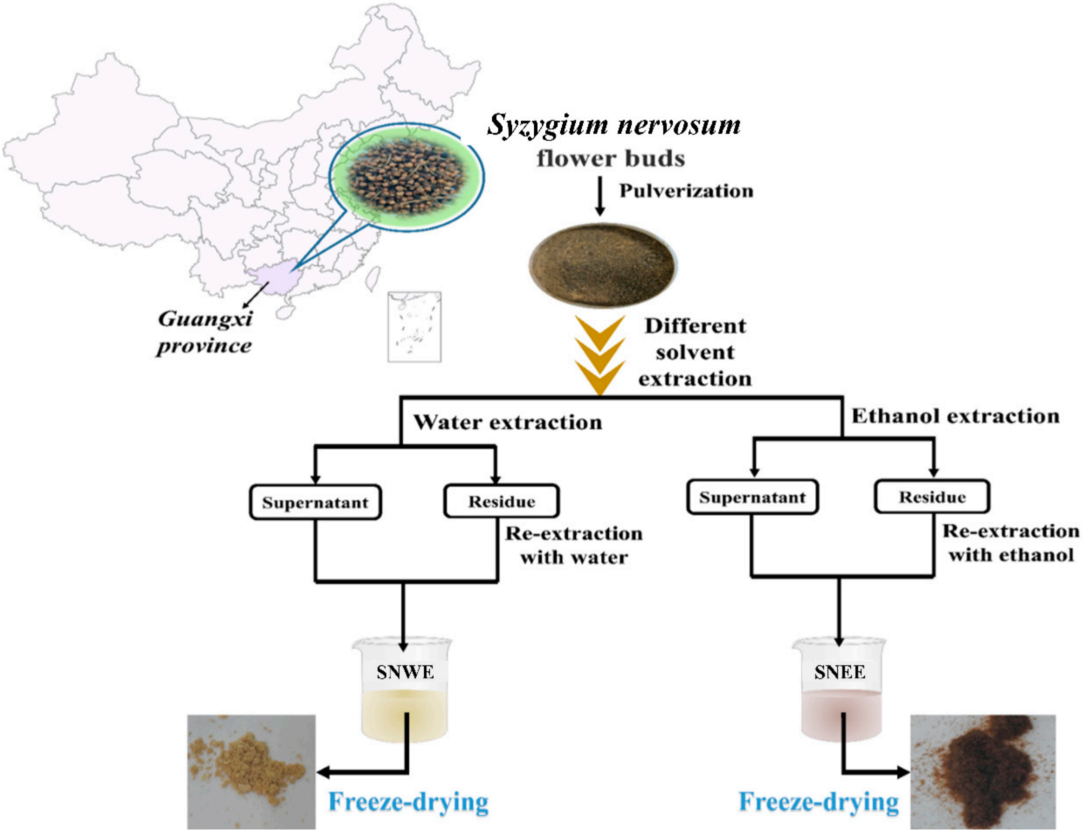

Figure S1. The schematic diagram of various solvent extraction processes applied to *Syzygium nervosum* flower buds

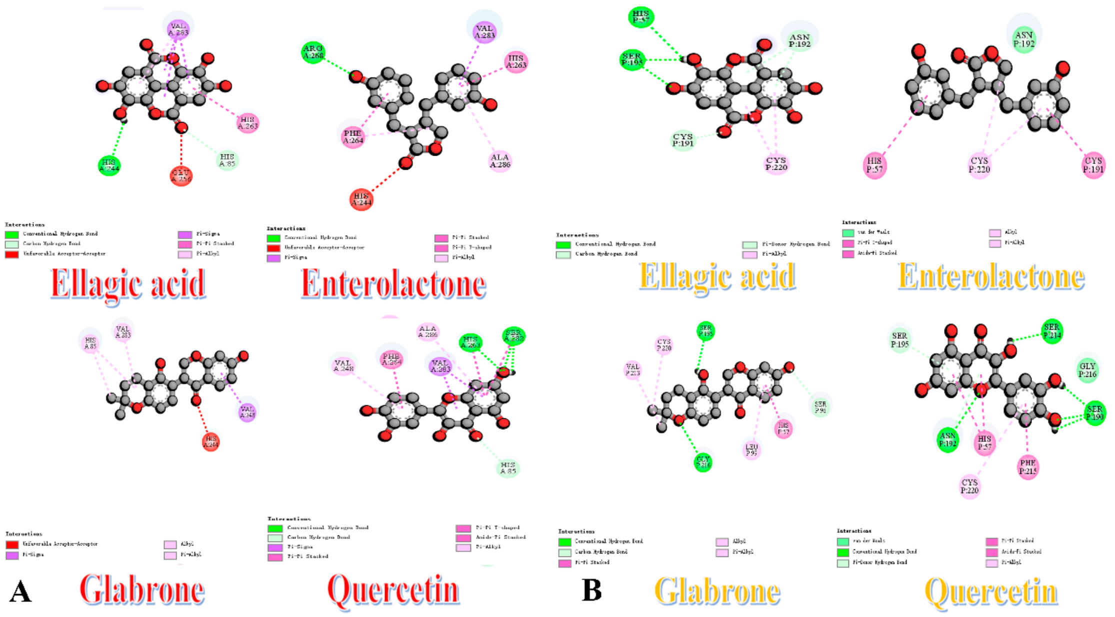

Figure S2. The 2D schematic interaction diagram between ellagic acid, enterolactone, glabrone, and quercetin and the active amino acid residues of the tyrosinase (A) and elastase (B).

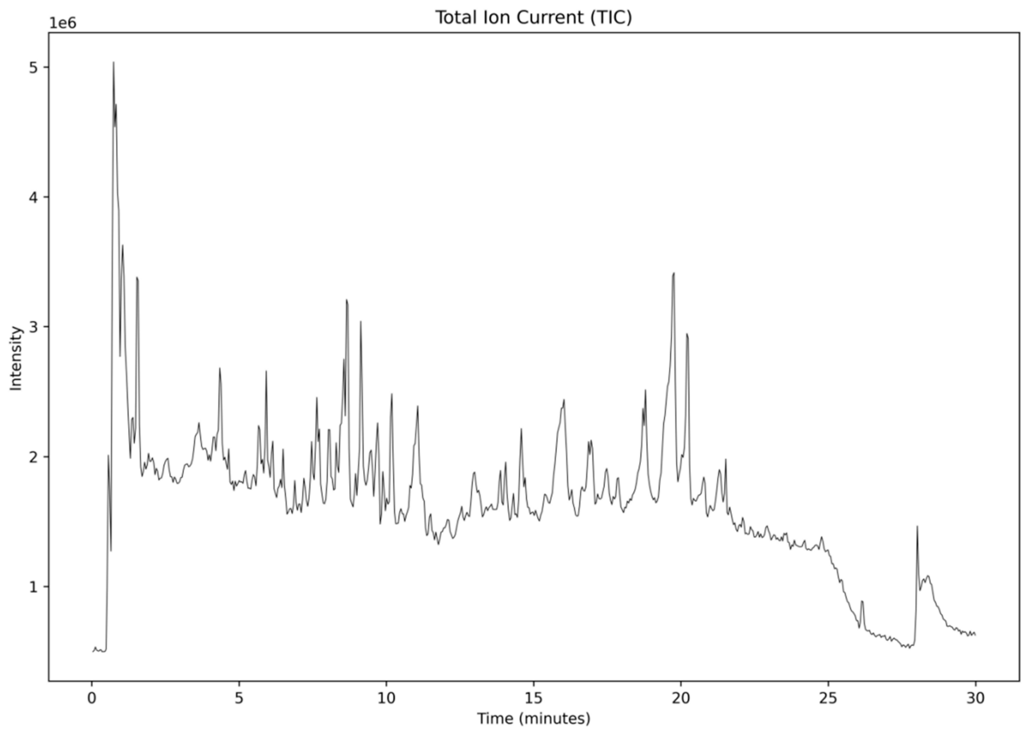

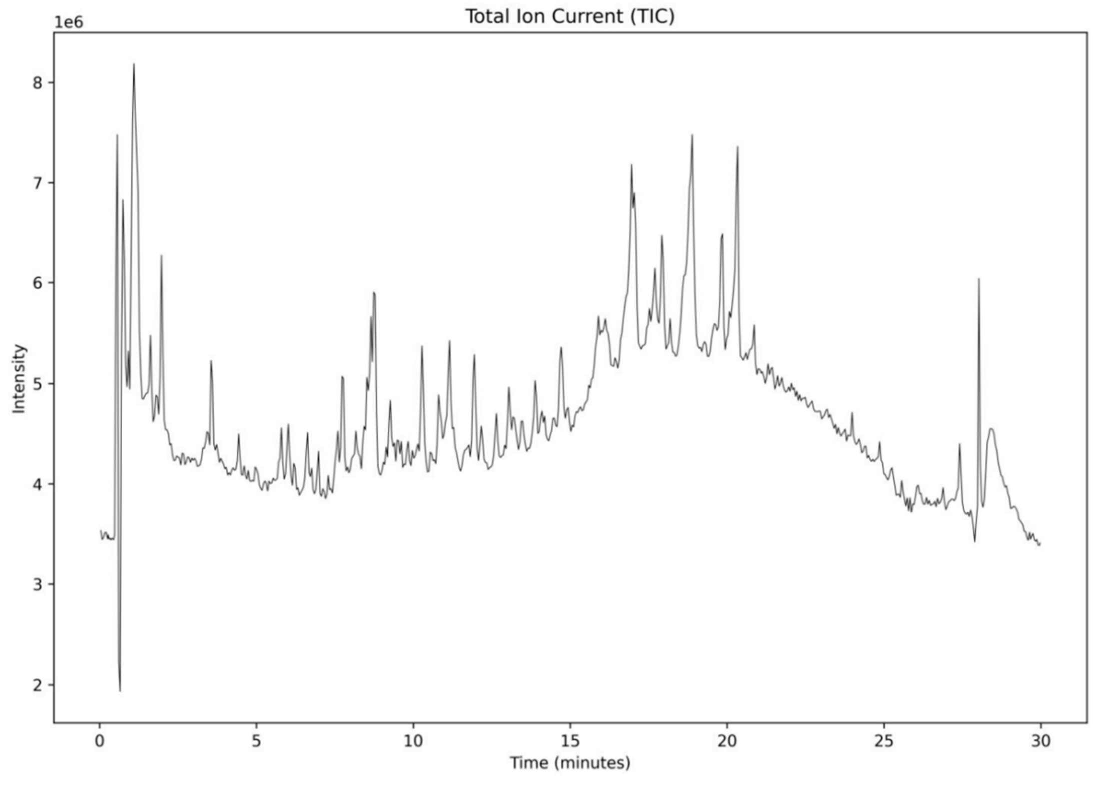

Figure S3. The total ion current (TIC) chromatogram of *Syzygium nervosum* extracts.

Table S1. Total phenolic and flavonoid contents of *Syzygium nervosum* extracts.

**Samples**	**Extraction yields (%)**	**Total phenolic contents (mg GAE/g of extract)**	**Total flavonoid contents (mg QE/g of extract)**
SNWE	12.09 ± 0.037	94.24 ± 0.613	26.67 ± 0.357
SNEE	7.13 ± 0.035	53.30 ± 0.474	18.00 ± 0.375

Table S2. Phytochemical compounds identified in the ethanol extract of *S. nervosum* flower buds via LC-Q-TOF-MS.

Number	RT[a] (min)	Component name	Molecular formula	*m/z*	Fragment	Total score
1	0.697	Gluconic acid	$C_6H_{12}O_7$	195.0535	75	83.7
2	0.697	Lactic acid	$C_3H_6O_3$	89.02583	66.7	79.6
3	0.697	Mannitol	$C_6H_{14}O_6$	181.074	57.7	79.8
4	0.697	D-glyceric acid	$C_3H_6O_4$	105.0208	60	62.4
5	0.697	5-Methoxypsoralen	$C_{12}H_8O_4$	215.0354	57.1	62.9
6	0.697	Hypoxanthine	$C_5H_4N_4O$	135.0315	50	61.6
7	0.698	Choline	$C_5H_{13}NO$	104.1083	60	81.8
8	0.741	Quinic acid	$C_7H_{12}O_6$	191.0586	89.3	89.5
9	0.741	Mannose	$C_6H_{12}O_6$	179.0584	72.7	78.5
10	0.742	Trigonelline	$C_7H_7NO_2$	138.055	75	91.4
11	0.742	Betaine	$C_5H_{11}NO_2$	118.0863	100	89.8
12	0.785	Mucic acid	$C_6H_{10}O_8$	209.0334	95.2	93.7
13	0.785	3(2'-Chlorophenyl)-7-hydroxy-4-phenylcoumarin	$C_{21}H_{13}ClO_3$	347.0518	100	74.1
14	0.873	Malic acid	$C_4H_6O_5$	133.016	100	85.7
15	0.873	Tartaric acid	$C_4H_6O_6$	149.0111	75	82.5
16	0.873	Maleic acid	$C_4H_4O_4$	115.0048	100	70.5

Number	RT[a] (min)	Component name	Molecular formula	*m/z*	Fragment	Total score
17	0.961	Uridine monophosphate	$C_9H_{13}N_2O_9P$	323.0324	75	73.8
18	1.005	Nicotinic acid	$C_6H_5NO_2$	124.0401	50	75.4
19	1.005	Triacanthine	$C_{10}H_{13}N_5$	204.1325	100	62.2
20	1.049	Uracil	$C_4H_4N_2O_2$	113.0345	66.7	78.4
21	1.092	Citric acid	$C_6H_8O_7$	191.0237	90	94.5
22	1.092	Trans-Aconitic acid	$C_6H_6O_6$	173.0114	100	64.3
23	1.093	Adenosine	$C_{10}H_{13}N_5O_4$	268.1047	100	83.3
24	1.093	Trans-ortho-coumaric acid	$C_9H_8O_3$	165.0548	50	61.5
25	1.180	Succinic acid	$C_4H_6O_4$	117.0213	80	76.6
26	1.180	Ascorbic acid	$C_6H_8O_6$	175.0275	53.8	67.1
27	1.181	Guanosine	$C_{10}H_{13}N_5O_5$	284.0995	100	85.7
28	1.181	Guanine	$C_5H_5N_5O$	152.0569	100	82.2
29	1.181	Piperidine	$C_5H_{11}N$	86.09641	100	64
30	1.224	Tiglic acid	$C_5H_8O_2$	99.04665	42.9	68.5
31	1.400	Ginkgetin	$C_{32}H_{22}O_{10}$	565.1126	50	60.3
32	1.532	Hydroxyhydroquinone	$C_6H_6O_3$	125.0258	73.3	90.6
33	1.532	Gallic acid	$C_7H_6O_5$	169.0177	100	89.5
34	1.532	3,4-	$C_8H_8O_4$	167.0007	50	63

Number	RT[a] (min)	Component name	Molecular formula	*m/z*	Fragment	Total score
		Dihydroxyphenyl acetic acid				
35	1.664	Glutaric acid	$C_5H_8O_4$	131.0371	75	71.8
36	1.972	Phenylalanine	$C_9H_{11}NO_2$	166.0877	60	86.6
37	2.498	Hesperetin	$C_{16}H_{14}O_6$	301.0642	100	70
38	2.938	1,2-benzenediol	$C_6H_6O_2$	109.0303	100	88.2
39	2.938	Protocatechuic acid	$C_7H_6O_4$	153.0212	100	83.5
40	2.981	Isobutyric acid	$C_4H_8O_2$	87.0461	100	73.9
41	2.982	Epigallocatechin	$C_{15}H_{14}O_7$	307.0821	100	83.9
42	2.982	4-hydroxybenzoic acid	$C_7H_6O_3$	139.0393	41.7	64.5
43	3.289	Iretol	$C_7H_8O_4$	155.0373	66.7	76.4
44	3.728	Diffractaic Acid	$C_{20}H_{22}O_7$	373.1175	57.1	61.4
45	3.728	Veratric acid	$C_9H_{10}O_4$	181.0518	100	66.7
46	4.081	Alnustone	$C_{19}H_{18}O$	263.139	100	65.3
47	4.168	Salicylic acid	$C_7H_6O_3$	137.0261	100	88.9
48	4.343	Coumaric acid	$C_9H_8O_3$	163.0424	66.7	73.3
49	4.387	DL-3,4-Dihydroxymandelic acid	$C_8H_8O_5$	183.0324	60	63
50	4.432	7-methoxy-6-	$C_{15}H_{18}O_6$	295.1253	100	61.1

Number	RT[a] (min)	Component name	Molecular formula	*m/z*	Fragment	Total score
		(1,2,3-trihydroxy-3-methylbutyl)chromen-2-one				
51	4.475	Gentisic acid	$C_7H_6O_4$	153.0206	100	86
52	4.475	Catechin	$C_{15}H_{14}O_6$	289.0757	85.2	80.2
53	4.608	Epicatechin	$C_{15}H_{14}O_6$	291.0872	43.8	73.9
54	4.740	Vanillic acid	$C_8H_8O_4$	169.0498	45	65
55	4.914	6,7-Dihydroxycoumarin	$C_9H_6O_4$	177.0215	75	73.5
56	5.047	Homogentisic acid	$C_8H_8O_4$	169.0499	42.9	63.4
57	5.178	Benzoic acid	$C_7H_6O_2$	121.0312	85.7	90.2
58	5.398	Syringic acid	$C_9H_{10}O_5$	197.048	77.8	66.2
59	5.529	Isookanin-7-O-glucoside	$C_{21}H_{22}O_{11}$	449.1137	77.8	66.4
60	5.750	Riboflavin	$C_{17}H_{20}N_4O_6$	377.1472	60	60.6
61	5.750	Bavachin	$C_{20}H_{20}O_4$	325.1406	50	68.9
62	5.794	Irigenin	$C_{18}H_{16}O_8$	361.0907	100	83.8
63	5.794	5,7-dihydroxy-4-methylcoumarin	$C_{10}H_8O_4$	193.0502	100	82.3

Number	RT[a] (min)	Component name	Molecular formula	*m/z*	Fragment	Total score
64	5.794	Coumarin	$C_9H_6O_2$	147.0441	66.7	70.9
65	5.837	Epigallocatechin-3-Monogallate	$C_{22}H_{18}O_{11}$	457.0832	70	61.3
66	6.014	trans-Cinnamic acid	$C_9H_8O_2$	149.0603	83.3	81.4
67	6.188	1,3,6-tri-O-galloylglucose	$C_{27}H_{24}O_{18}$	635.0941	72.7	70.3
68	6.188	Epigallocatechin-3-gallate	$C_{22}H_{18}O_{11}$	457.0821	58.8	63
69	6.189	4-Hydroxy-3-methoxycinnamaldehyde	$C_{10}H_{10}O_3$	179.071	50	61.3
70	6.189	Chlorogenic Acid	$C_{16}H_{18}O_9$	355.1041	100	61.5
71	6.408	Trans-4-Coumaric acid	$C_9H_8O_3$	163.0422	100	86.3
72	6.452	Quercetin-3,4'-O-di-beta-glucoside	$C_{27}H_{30}O_{17}$	625.147	60	60.7
73	6.496	Myricetin-3-O-galactoside	$C_{21}H_{20}O_{13}$	479.088	85.7	89.7
74	6.629	Myricetin-3-O-hexoside	$C_{21}H_{20}O_{13}$	481.0992	60	88.1
75	7.155	Myricetin-3-O-	$C_{20}H_{18}O_{12}$	449.0776	100	74.3

Number	RT[a] (min)	Component name	Molecular formula	*m/z*	Fragment	Total score
		xyloside				
76	7.288	Myricetin	$C_{15}H_{10}O_8$	319.0461	50	82
77	7.331	1,2,3,6-tetragalloylglucose	$C_{34}H_{28}O_{22}$	787.1085	85.7	74.7
78	7.332	Naringenin	$C_{15}H_{12}O_5$	273.0765	42.9	65
79	7.507	3'-hydroxyPuerarin	$C_{21}H_{20}O_{10}$	433.114	50	67.6
80	7.594	Ellagic acid	$C_{14}H_6O_8$	301.0025	75	83.8
81	7.595	Isoquercitrin	$C_{21}H_{20}O_{12}$	465.1038	100	91
82	7.595	3',4',5,5',7-pentahydroxyflavone	$C_{15}H_{10}O_7$	303.0504	100	85.1
83	7.726	Theophylline	$C_7H_8N_4O_2$	179.0582	100	68.5
84	8.033	3,4-Dihydroxymandelic acid	$C_8H_8O_5$	183.0302	80	67.8
85	8.033	Sinapic acid	$C_{11}H_{12}O_5$	223.0637	100	63.3
86	8.077	6-O-p-Coumaroyl-1,2-digalloylglucose	$C_{29}H_{26}O_{16}$	629.1212	40.6	63.3
87	8.165	Astragalin	$C_{21}H_{20}O_{11}$	447.0978	100	89.4

Number	RT[a] (min)	Component name	Molecular formula	*m/z*	Fragment	Total score
88	8.254	Reynoutrin	$C_{20}H_{18}O_{11}$	435.0937	100	92.5
89	8.297	Avicularin	$C_{20}H_{18}O_{11}$	433.0822	100	85.2
90	8.430	Quercetin	$C_{15}H_{10}O_{7}$	303.0508	100	81.6
91	8.561	Isorhamnetin-3-glucoside	$C_{22}H_{22}O_{12}$	477.1088	100	90.5
92	8.648	Syringetin-3-O-glucoside	$C_{23}H_{24}O_{13}$	507.1205	100	90
93	8.650	Isorhamnetin	$C_{16}H_{12}O_{7}$	317.0666	62.5	87.4
94	8.650	Isorhamnetin-3-O-beta-D-Glucoside	$C_{22}H_{22}O_{12}$	479.1209	100	92.5
95	8.692	Azelaic acid	$C_{9}H_{16}O_{4}$	187.1	77.8	84.3
96	8.738	Limocitrin	$C_{17}H_{14}O_{8}$	347.0779	100	89.9
97	8.824	3-alpha-L-arabinopyranoside	$C_{20}H_{18}O_{10}$	417.0878	80	72.7
98	8.868	Dihydrokaempferol	$C_{15}H_{12}O_{6}$	287.0598	52	68.8
99	9.177	Chrysoeriol 7-O-glucoside	$C_{22}H_{22}O_{11}$	463.1247	75	85.7
100	9.264	Luteolin-7-O-glucoside	$C_{21}H_{20}O_{11}$	447.0981	100	73.7
101	9.265	Phloretin	$C_{15}H_{14}O_{5}$	275.0925	50	66.2

Number	RT[a] (min)	Component name	Molecular formula	*m/z*	Fragment	Total score
102	9.397	Liquiritigenin	$C_{15}H_{12}O_4$	257.081	100	61.4
103	9.616	Sinapyl alcohol	$C_{11}H_{14}O_4$	211.0967	100	62.9
104	9.703	Liquiritin	$C_{21}H_{22}O_9$	417.1237	100	76.2
105	9.792	(-)-Eburnamonine	$C_{19}H_{22}N_2O$	295.1887	100	73.2
106	10.494	Abscisic Acid	$C_{15}H_{20}O_4$	263.133	83.3	78.7
107	10.539	Gardenin B	$C_{19}H_{18}O_7$	359.1115	100	69.8
108	10.581	Mycophenolic acid	$C_{17}H_{20}O_6$	319.1216	100	64.3
109	10.713	Sebacic acid	$C_{10}H_{18}O_4$	201.1154	62.5	68.4
110	10.802	Mulberroside A	$C_{26}H_{32}O_{14}$	569.1811	100	66.9
111	10.845	5,6-Dihydroxy-3',4'-dimethoxyflavanone	$C_{17}H_{16}O_6$	315.0898	72.7	61
112	11.021	3',4',5,7-tetrahydroxyflavone	$C_{15}H_{10}O_6$	285.0437	92.3	84.5
113	11.066	(2E)-3-(4-hydroxy-3-methoxyphenyl)-2-propenoic acid	$C_{10}H_{10}O_4$	195.0655	100	81
114	11.330	3-methylquercetin	$C_{16}H_{12}O_7$	317.0669	100	85.5

Number	RT[a] (min)	Component name	Molecular formula	*m/z*	Fragment	Total score
115	11.373	Methyl 2-((4-methyl-2-oxo-2H-chromen-7-yl)oxy)propanoate	$C_{14}H_{14}O_5$	263.092	100	72
116	11.461	Dihydrocapsaicin	$C_{18}H_{29}NO_3$	308.2225	100	61
117	11.945	Glabrone	$C_{20}H_{16}O_5$	337.1052	100	71.3
118	12.208	Carvone	$C_{10}H_{14}O$	151.1118	43.8	70.6
119	12.734	3'-methoxy-4',5,7-trihydroxyflavonol	$C_{16}H_{12}O_7$	315.0549	100	74.5
120	12.734	Kaempferol	$C_{15}H_{10}O_6$	285.0442	100	69.1
121	12.822	Methyl vanillate	$C_8H_8O_4$	181.053	60	67.6
122	12.911	Harmine	$C_{13}H_{12}N_2O$	213.1028	100	69.6
123	12.954	Sorbifolin	$C_{16}H_{12}O_6$	299.0597	80	72.4
124	13.086	Xanthoxylin	$C_{10}H_{12}O_4$	195.0676	45	69.3
125	13.087	Tectorigenin	$C_{16}H_{12}O_6$	301.0713	100	84.9
126	13.087	Vinpocetine	$C_{22}H_{26}N_2O_2$	351.2147	50	65.5
127	13.087	Jaceosidin	$C_{17}H_{14}O_7$	331.0825	100	74.7
128	13.131	isopsoralidin	$C_{20}H_{16}O_5$	337.1082	100	61.2
129	13.263	licoflavanone	$C_{20}H_{20}O_5$	341.1396	50	68.1
130	13.438	Laetanine	$C_{18}H_{19}NO_4$	314.1391	60	61.5
131	13.482	Luvangetin	$C_{15}H_{14}O_4$	259.0969	100	60.4

Number	RT[a] (min)	Component name	Molecular formula	*m/z*	Fragment	Total score
132	13.790	3-Acetylaconitine	$C_{36}H_{49}NO_{12}$	688.3269	50	71.4
133	14.185	L-Asarinin	$C_{20}H_{18}O_6$	355.1165	100	77.3
134	14.361	Quinine	$C_{20}H_{24}N_2O_2$	325.1985	100	69.5
135	14.491	Uridine	$C_9H_{12}N_2O_6$	243.0675	50	66.9
136	14.491	Atractylenolide III	$C_{15}H_{20}O_3$	247.1357	66.7	66.7
137	14.493	Stepharine	$C_{18}H_{19}NO_3$	298.1445	100	81.3
138	14.668	Ethyl caffeate	$C_{11}H_{12}O_4$	209.0813	100	70.6
139	14.799	Madecassic acid	$C_{30}H_{48}O_6$	503.3429	100	72.6
140	15.240	Rotenone	$C_{23}H_{22}O_6$	395.1522	100	70.8
141	15.326	3,5-dimethoxyphenol	$C_8H_{10}O_3$	153.0577	50	72.6
142	15.591	Flavokawain b	$C_{17}H_{16}O_4$	285.1134	66.7	64.8
143	15.634	Arctigenin	$C_{21}H_{24}O_6$	371.1532	100	60.7
144	16.073	Cardamonin	$C_{16}H_{14}O_4$	269.0854	50	66.4
145	16.293	Farrerol	$C_{17}H_{16}O_5$	299.0955	50	71.5
146	16.426	Licoflavone C	$C_{20}H_{18}O_5$	339.1233	50	75.1
147	16.864	6-Ethoxy-3(4'-hydroxyphenyl)-4-methylcoumarin	$C_{18}H_{16}O_4$	295.1008	100	73.1
148	17.435	Asiatic acid	$C_{30}H_{48}O_5$	487.3481	60	76.5
149	18.313	DMC	$C_{18}H_{18}O_4$	297.0808	66.7	64

Number	RT[a] (min)	Component name	Molecular formula	*m/z*	Fragment	Total score
150	18.533	8-Prenylnaringenin	$C_{20}H_{20}O_5$	339.1264	50	64.4
151	18.578	5,7-dimethoxy-2-phenyl-4H-chromen-4-one	$C_{17}H_{14}O_4$	283.0974	100	81.2
152	18.709	Phenethyl Caffeiate	$C_{17}H_{16}O_4$	283.1007	80	83.6
153	18.798	5,7-Dimethoxyflavanone	$C_{17}H_{16}O_4$	285.113	50	84.4
154	18.886	Corylin	$C_{20}H_{16}O_4$	321.1107	100	83
155	19.018	Beta-Boswellic acid	$C_{30}H_{48}O_3$	457.3696	100	62.9
156	19.675	Enterolactone	$C_{18}H_{18}O_4$	297.1192	66.7	81.1
157	20.248	Linolenic acid	$C_{18}H_{30}O_2$	279.2327	41.2	63.9
158	20.380	Bayogenin	$C_{30}H_{48}O_5$	489.3581	66.7	73.3
159	20.642	Glycyrrhetinic Acid	$C_{30}H_{46}O_4$	469.3372	100	72
160	20.687	Isoxanthohumol	$C_{21}H_{22}O_5$	355.1549	50	63.2
161	20.773	Hederagenin	$C_{30}H_{48}O_4$	471.3531	100	79.6
162	21.345	4-Octylphenol	$C_{14}H_{22}O$	205.161	100	63.6
163	21.390	Dibutyl phthalate	$C_{16}H_{22}O_4$	279.1595	50	79.7

Number	RT[a] (min)	Component name	Molecular formula	*m/z*	Fragment	Total score
164	21.872	Osajin	$C_{25}H_{24}O_5$	403.1591	50	63.6
165	22.049	Emetine Dihydrochloride	$C_{29}H_{42}Cl_2N_2O_4$	553.2574	50	61
166	22.269	Saikogenin D	$C_{30}H_{48}O_4$	473.3629	50	65.9
167	23.585	Ursolic acid	$C_{30}H_{48}O_3$	455.3573	50	77
168	25.564	Rescinnamine	$C_{35}H_{42}N_2O_9$	635.2896	50	74.6
169	26.177	Stearic acid	$C_{18}H_{36}O_2$	283.2654	100	78.1
170	26.177	Isorhynchophylline	$C_{22}H_{28}N_2O_4$	383.1942	100	72.8
171	27.321	Coniine	$C_8H_{17}N$	128.1434	50	83.2

RT[a] – Retention time.
